# Supplementary material for: HIV prevalence, sexual risk behaviours and HIV testing among female sex workers in three cities in Sri Lanka: Findings from respondent-driven sampling surveys
Source: PLoS One. 2020 Oct 1;15(10):e0239951. doi: 10.1371/journal.pone.0239951 (PMC7529248; doi:10.1371/journal.pone.0239951)
Supplement: Attachment — Submitted filename: Response to reviewers.docx [file pone.0239951.s004.docx]

**Responses to comments of the Editor and reviewers**

PONE-D-20-14017
HIV prevalence, sexual risk behaviours and HIV testing among female sex workers in three cities in Sri Lanka: Findings from respondent-driven sampling surveys

| **Comments of the Editorial Office** | Responses of the authors |
| --- | --- |
| Please ensure that your manuscript meets PLOS ONE's style requirements, including those for file naming | Thank you, this has been corrected. |
| Please amend your current ethics statement to address the following concerns: Please explain why written consent was not obtained, how you recorded/documented participant consent, and if the ethics committees/IRBs approved this consent procedure. | Written consent was not obtained as it is a standard practice in HIV bio-behavioural surveys in key populations that written consent is not asked for, but an oral consent. Following is added:  After they gave an oral consent to participate, a survey staff member signed an informed consent form on their behalf. The Ethics Committee approved this consent procedure. |
| Please provide more information on the coupons given out as part of recruitment. | More information is now provided in Methods:  Each coupon had its unique number that enabled to link participants with those who gave them coupons to participate in the study. Coupon numbers enable to monitor recruitment chains and issue secondary incentives, and are also necessary for RDS data analysis. Coupons also contained information about the address of the study site and time-period when the study site is open. |
| We note that you have indicated that data from this study are available upon request. PLOS only allows data to be available upon request if there are legal or ethical restrictions on sharing data publicly. | There are no legal or ethical restrictions on sharing these data publicly.  We will upload the anonymized data set necessary to replicate your study finding. |
| **Reviewer 1** |  |
| Major: Could the author explain the reason for choosing the three cities as the research site? How representative are the results of these three cities? | These three cities were selected for IBBS in 2018 because IBBS were done in 2015 in these three cities. By repeating surveys in the same cities over time, changes in HIV indicators over time can be assessed. These three cities were selected in 2015 (and 2018) because they are in the most populous provinces in Sri Lanka named Western Province (Colombo), Central Province (Kandy), and Southern Province (Galle) and because of estimated population size of FSW.  This explanation is added at the end of Introduction. |
| Minor: 1.There is a lack of background data on sexual risk behaviors and HIV testing among female sex workers in Sri Lanka in the introduction part. Please provide more data if possible.  2.Please clarify the reason for choosing “consistent condom use with a non-paying partner for 30 days” as the indicator in sample size estimation.  3.Please add more details of the measurement of Sexual risk behaviors since it is one of the major outcomes.  4.Please clarify why only the result of Colombo was weighted in table 1.  5.Please provide the statistical testing results (like P-value) in the comparison between two rounds of the survey in different cities in table 2. (2015 versus 2018)  6.Please mark the significance of aOR in table 3. | 1. Thank you, more data are added on sexual risk behaviours and HIV testing in Introduction.  2. We chose this indicator because this is an important indicator of sexual behaviours in FSW. We could not base sample size calculation on prevalence of infections because of low prevalence estimates from IBBS in FSW in 2015.  3. This is added now to Methods  4. All prevalence estimates were weighted. This is corrected now in Table 1.  5. We compared now all indicators collected in the 2015 and 2018 surveys using chi-square test, and denoted with a symbol * in Table 2 where there is significant differences (p<0.05) in values.  6. This is now marked with an * |
| Reviewer 2 |  |
| 1. In the introduction, the authors should introduce why they chose the three places (Colombo, Galle and Kandy) as the study sites?  2. The authors should provide a further implication of this study, not only to monitor and further characteristics HIV-related risk behaviors, uptake of HIV prevention services and HIV prevalence? | 1. This is now added to the Introduction.  2. We provided several implications for programmatic interventions, in particular need to expand community-based HIV testing, address financial and social vulnerabilities and focus on empowerment-based approaches.  We added now a need for interventions to focus on promoting consistent condom use across all types of partnerships and increase skills in negotiating condom use. |
| 1. Is the FSW collected in 2015 and 2018 the same group? Is there any difference between the FSW in 2015 and 2018? Could the authors add the data of 2015 and compared with that of 2018 in Table 1 for the demographic characteristics?  2. The unit of age in Table 1 should be added.  3. Can you rearrange the contents of Table 2 logically, which is very difficult to read. Also, can you compare the knowledge about HIV, sexual behaviors and health care utilization between 2015 and 2018?  4. If the author compares the differences between the three regions, more information may be provided. | 1. We added some demographic data on FSW recruited in 2015 to the paragraph in Results under socio-demographic characteristics. We thought that this is better than adding data to Table 1.  2.This is added to Table 1  3. We re-arranged the contents of Table 2 so that it is more logical.  We compared now all indicators collected in the 2015 and 2018 survey using chi-square test, and denoted with a sign of * in the Table 2 where there is significant differences (p<0.05)  4. We provided more information on socio-economic differences between Colombo, Galle and Kandy in Introduction. This information was collected in DHS in 2016. |
| Discussion  The author should add more information about the three study sites of social economics, which could give more information to explain the differences of factors associated not being tested for HIV in the past 12 months or not knowing HIV positive status among FSW.  2. Selection bias may be a limitation in this study. | As stated above, we provided more information on socio-economic differences between Colombo, Galle and Kandy in Introduction. This information was collected in DHS in 2016.  Implications of these data is discussed in Discussion.  2. Agree, this is added to Limitations. |
|  |  |
