# Supplementary material for: HIV prevalence, sexual risk behaviours and HIV testing among female sex workers in three cities in Sri Lanka: Findings from respondent-driven sampling surveys
Source: PLoS One. 2020 Oct 1;15(10):e0239951. doi: 10.1371/journal.pone.0239951 (PMC7529248; doi:10.1371/journal.pone.0239951)
Supplement: Attachment — Submitted filename: Response to reviewers.docx [file pone.0239951.s005.docx]

**Responses to comments of the reviewers**

**28 August 2020**

PONE-D-20-14017
HIV prevalence, sexual risk behaviours and HIV testing among female sex workers in three cities in Sri Lanka: Findings from respondent-driven sampling surveys

| **Comments of Reviewer 1** | Responses of the authors |
| --- | --- |
| 1. Sample size calculation: the author explained in the last response that using ‘condom use with a non-paying partner’ to calculate sample size was due to the importance of condom use, however, there are several behavioral indicators including testing for HIV/knowing one’s status, which is the outcome of the regression. It would be better if the author could calculate sample sizes with other indicators and compare them with the current sample size to demonstrate the power of the current choice. If other calculated sample sizes were larger, limitations should be addressed in discussion. | We did the calculation of sample sizes for several other indicators including “tested for HIV in the past 12 months or knowing one’s status” and “comprehensive knowledge about HIV prevention” but that yielded smaller sample sizes compared to ‘condom use with a non-paying partner’.  Used condom at last sex with a client was not used to calculate the sample size as the value of this indicator was already high in the 2015 IBBS, so a large sample size would have been needed to detect a small change in this indicator.  Final samples sizes that we calculated and the number of FSW recruited - 458 FSW in Colombo, 360 in Galle and 362 in Kandy – is comparable to IBBS done in KPs in other settings. |
| 2. The outcome: First, the outcome of regression is “not testing or not knowing one’s status”, yet in table 2, there was one variable named “tested or know”. It would be good if the author can clarify if the rates of these two variables are complementary and the sum of them is 1. If so, should the outcome of regression be “not testing and not knowing”? Please ensure the accuracy of the expression.  Second, as was mentioned in the discussion, the rate of “Recent HIV testing” ranged from 17.2% to 39.5%. However, after reading table 2 carefully, the variables that match this expression is “tested for HIV in the past 12 months or knowing HIV positive status”. Though the HIV positive rates were low in this study, it was not appropriate to mix these two indicators in the abstract and discussion. Please revise.  Third, the number of 17.2% in the second suggestion wasn’t displayed in table2. The closest number was 17.1 and 17.5. Please make sure there is no typo. | The outcome of regression is “not being tested for HIV in the past 12 months or not knowing HIV positive status”. This is in the title of the Table 3.  Now, we added this clarification to the the columns of Table 3.  In Table 2, the outcome is “Tested for HIV in the past 12 months or knowing HIV positive status”.  The sum of these proportions is 1.  This is right, we corrected this so that instead of “recent HIV testing” it stands “tested for HIV in the past 12 months or knowing HIV positive status”.  It should be 17.5% - this is now corrected in Abstract and Discussion |
| 3. The discussion: The author provided statistical testing between data in 2015 and 2018. And there were many significant results, which haven’t been referred to or deeply discussed in the discussion part. It would be better if the author could provide more insight into the reason of changes from 2015 to 2018, as well as implication for policy makers. | We provided a discussion about significant differences in some results, in particular HIV testing, condom use and comprehensive knowledge about HIV prevention.  The implication of results in terms of development of HIV response in FSW is addressed later on in Discussion. |
| Uses with dots and commas: In paragraph one of introduction, both line one and line five have displayed a confusing mixture of dots and commas. Please proofread the paper and make sure such uses are consistent within the manuscript. | This was corrected in the Introduction, and checked in other parts of the manuscript. |
| **Reviewer 2** |  |
| Introduction: In the last paragraph, the author should write the research hypothesis rather than the results of the demographic and health survey. | Research hypothesis was added to the Introduction (We hypothesised that there will be a significant increase in condom use with non-paying partners and ever testing for HIV when 2015 data are compared with 2018.)  During the first round of review, the other reviewer suggested to add results on demographics in these three cities. |
| In Table 2  The first column can be classified by knowledge, behavior and health care utilization. | This is added now to classify indicators |
